# Set anode potentials affect the electron fluxes and microbial community structure in propionate-fed microbial electrolysis cells

**DOI:** 10.1038/srep38690

**Published:** 2016-12-09

**Authors:** Ananda Rao Hari, Krishna P. Katuri, Bruce E. Logan, Pascal E. Saikaly

**Affiliations:** 1 King Abdullah University of Science and Technology, Biological and Environmental Sciences and Engineering Division, Water Desalination and Reuse Research Center, Thuwal, 23955-6900, Saudi Arabia; 2 The Pennsylvania State University, Department of Civil and Environmental Engineering, University Park, PA 16802, USA

## Abstract

Anode potential has been shown to be a critical factor in the rate of acetate removal in microbial electrolysis cells (MECs), but studies with fermentable substrates and set potentials are lacking. Here, we examined the impact of three different set anode potentials (SAPs; −0.25, 0, and 0.25 V vs. standard hydrogen electrode) on the electrochemical performance, electron flux to various sinks, and anodic microbial community structure in two-chambered MECs fed with propionate. Electrical current (49–71%) and CH_4_ (22.9–41%) were the largest electron sinks regardless of the potentials tested. Among the three SAPs tested, 0 V showed the highest electron flux to electrical current (71 ± 5%) and the lowest flux to CH_4_ (22.9 ± 1.2%). In contrast, the SAP of −0.25 V had the lowest electron flux to current (49 ± 6%) and the highest flux to CH_4_ (41.1 ± 2%). The most dominant genera detected on the anode of all three SAPs based on 16S rRNA gene sequencing were *Geobacter, Smithella* and *Syntrophobacter*, but their relative abundance varied among the tested SAPs. Microbial community analysis implies that complete degradation of propionate in all the tested SAPs was facilitated by syntrophic interactions between fermenters and *Geobacter* at the anode and ferementers and hydrogenotrophic methanogens in suspension.

Microbial electrochemical technologies (METs) are widely recognized for their potential for recovering energy from organic waste streams. In many different METs, certain microorganisms known as exoelectrogens convert chemical energy in organics to electricity via anaerobic oxidation of wastewater organics at the anode. The electrons and protons that are generated during oxidation at the anode are utilized at the cathode for oxygen reduction reaction in microbial fuel cells (MFCs) or hydrogen evolution reaction in microbial electrolysis cells (MECs)^1^. MECs can be operated either by setting the anode potential using a potentiostat, or applying voltage using an external power source^2^ or by inserting a reverse electrodialysis stack between the electrodes^3^. Setting different anode potentials can impact the electrochemical performance, microbial community structure and theoretical maximum energy gain (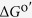) by exoelectrogens for their growth and maintenance^4^,^5^,^6^, according to:


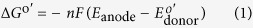


where 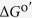 (J/mol) is the Gibbs free energy at standard biological conditions (T = 25 °C, pH = 7 and 1 M of all reactants), *n* the number of electrons transmitted, *F F*aradays constant (96,485 C/mol e^−^), *E*_anode_ the anode potential (V), and 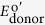 (V) the standard biological redox potential of the electron donor. The real energy gain by exoelectrogens depends on the redox potential of the terminal electron transferring component (e.g., outer membrane protein) serving as the electron donor to the anode^4^. In theory, at low anode potentials, exoelectrogens gain less energy for growth, resulting in lower biomass accumulation, slower development of the anode biofilm community, and more delayed start-up of current production^5^. At higher anode potentials, exoelectrogens could gain more energy for growth if they have the capability to capture this additional energy^4^.

One new and important application of MECs is the addition of electrodes directly into an anaerobic digester, in order to improve performance and increase the methane concentration in the product gas^7^,^8^,^9^,^10^. Such an integration is not practical using MFCs as the anaerobic digestion (AD) process requires oxygen free environment. However, the impact of a set anode potential (SAP) on MEC performance has not been well examined for such environments where there can be high concentrations of fermentable substrates, especially for the case of propionate which is slowly degraded, as most studies on SAPs in MECs have mainly focused on acetate^1^,^11^,^12^ (Supplementary Table S1). The performance of MECs is well known to be impacted in terms of current production for simple substrates such as acetate, although the community structure is relatively unchanged for different SAPs^1^. When SAPs have been examined in MFCs using fermentable substrates such as xylose, sucrose, formate, and glucose^6^,^13^,^14^,^15^, it was shown that the SAP influenced the biocatalytic activity, electrochemical performance, substrate degradation, and microbial community structure. Also, it altered the syntrophic interactions between the organisms^14^.

In METs fed fermentable substrates, methane generation by hydrogenotrophic methanogens is an important sink of electrons, and hence a coulombic loss^16^,^17^. The H_2_ generated during the fermentation process is utilized by hydrogenotrophic methanogens, which could outcompete other H_2_-utilizing organisms (e.g. exoelectrogens and homoacetogens), thus diverting electrons away from current^18^,^19^,^20^. The impact of various approches on methanogenesis has been examined in previous MEC studies such as the use of methanogen inhibitors^21^, reactor exposure to air^22^,^23^, the use of short hydraulic retention times^24^,^25^ and operating the reactors at low temperature of 15 °C^26^. However, the impact of different SAPs in MECs on the electron fluxes to methane versus current has not been examined with fermentable susbtrates.

Propionate is a model fermentable substrate to study microbial partnerships in natural and engineered methanogenic systems and an important intermediate in the anaerobic decomposition of organic matter in AD processes. Accumulation of propionate (>20 mM) at high organic loading rates is detrimental to AD processes^27^,^28^,^29^. Thus, propionate removal is necessary for the stable operation of AD. In these systems, propionate is oxidized via a microbial partnership between fermenters and methanogens. Fermenters oxidize propionate to acetate, formate/H_2_ and CO_2_, which are then utilized by methanogens to produce CH_4_. Recently, Hari *et al*. demonstrated that propionate degradation at the anode of MECs operated at an applied voltage of 0.7 V using a power source occurs via a microbial partnership between fermenters and *Geobacter*^30^. They showed that multiple paths of electron flow to current (via acetate/H_2_ or acetate/formate) could occur simultaneously during propionate oxidation regardless of the propionate concentration tested. At a high propionate concentration (36 mM), there was incomplete propionate removal (80%), compared to complete propionate removal at a much lower concentration (4.5 mM). While the use of an applied voltage was useful for studying MEC operation under these different conditions, the anode potentials in the reactors were not controlled, and thus the direct impact of the anode potential could not be determined in these studies separate from other operational conditions.

In order to better understand the impact of anode potential on a fermentable substrate, the effect of three different SAPs (−0.25, 0 and 0.25 V vs. standard hydrogen electrode, SHE) was examined on MEC performance using propionate in terms of its degradation rate, electron fluxes to various sinks (current, CH_4_ and undefined sinks), and microbial community structure using two-chamber reactors that minimized hydrogen gas crossover from the cathode.

## Results

### Electrochemical performance of MECs at different SAPs

**Figure 1:**
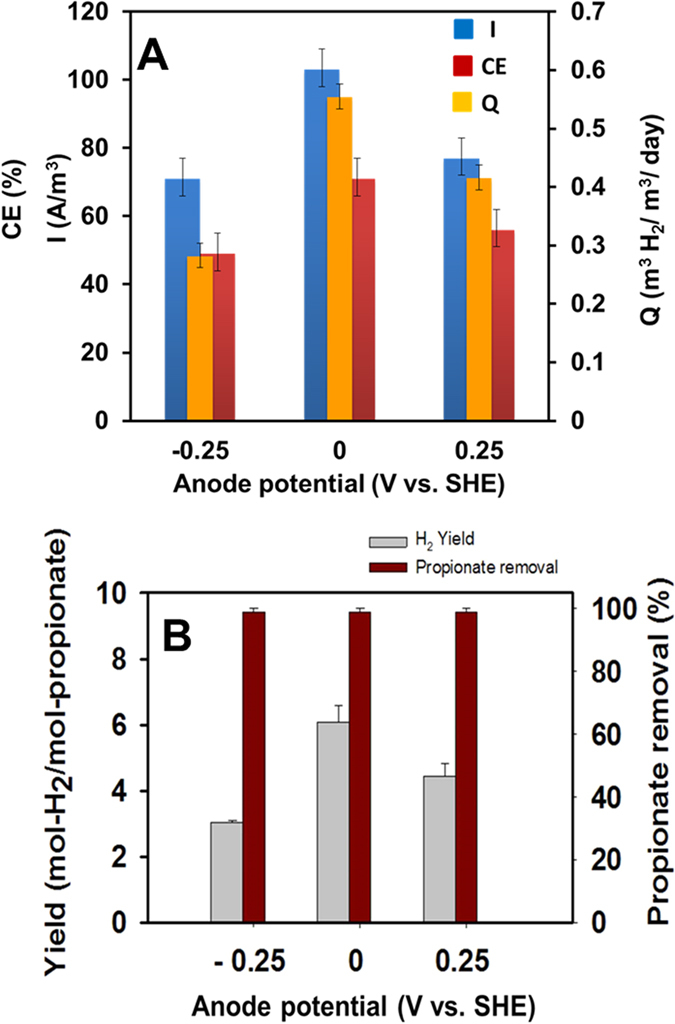
Performance of the MECs at different SAPs (−0.25, 0, and 0.25 V vs. SHE). (**A**) current density ((I) A/m^3^), coulombic efficiency ((CE) %) and H_2_ production rate ((Q) m^3^H_2_/m^3^/day). (**B**) H_2_ yield and propionate removal. Values represent the average of the last five batch cycles of the duplicate reactors.

MECs operated at SAP of 0 V performed significantly better than −0.25 and 0.25 V in terms of current density (average of maximum current density), CE, H_2_ production rates, and H_2_ yields (*P* < 0.05, student’s *t* test for all comparisons) (Fig. 1A,B). The values in Fig. 1 correspond to the average of the last five batch cycles of the duplicate reactors (*n* = 10). The peak current density was higher at 0 V (103 ± 5 A/m^3^) than −0.25 V (77 ± 5 A/m^3^) and 0. 25 V (71 ± 6 A/m^3^) by 25 ± 6% and 31 ± 5% respectively (*P* < 0.05) (Fig. 1A). The maximum current density (average of duplicate MECs) profile for all the batch cycles of operation is shown in Supplementary Figure S1. The ohmic drop compensation analysis (Supplementary Figure S2) using the peak current achieved at individual batch cycles (Supplementary Figure S1) and the carbon fiber brush anode resistance measured by electrochemical impedance spectroscopy, confirms that the ohmic drop (applied set potential versus measured potential of the anode) in the MECs operated at different SAPs was minimal. Similarly, CE was higher at 0 V (71 ± 5%), compared to −0.25 V (49 ± 6%) and 0.25 V (56 ± 5%) (*P* < 0.05) (Fig. 1A). H_2_ production rate was higher at 0 V (0.55 ± 0.01 m^3^ H_2_/m^3^/day) than 0.25 (0.42 ± 0.02 m^3^ H_2_/m^3^/day) and −0. 25 V (0.28 ± 0.02 m^3^ H_2_/m^3^/day) by 23% and 49% respectively (*P* < 0.05) (Fig. 1A). Although all the SAP-MECs showed nearly complete propionate degradation (99 ± 1%), the MECs operated at SAP of 0 V (5.90 ± 0.18 mol H_2_/mol propionate) showed significantly higher H_2_ yield than −0.25 (2.96 ± 0.21 mol H_2_/mol propionate) and 0.25 V (4.31 ± 0.19 mol H_2_/mol propionate) (*P* < 0.05) (Fig. 1B). The pH of the suspension of SAP-MECs and O.C reactors at the end of the batch was 7.8 ± 0.13 and 8.5 ± 0.2 respectively.

### Experimental distribution of electrons from propionate oxidation at the end of the last batch cycle

Electron balances at the end of the last batch cycle with matured biofilms (aged 155 days) indicated that electrical current was the first and CH_4_ was the second largest electron sinks among all the tested SAP conditions (Table 1). The SAP of 0 V showed higher electron distribution to electrical current (71 ± 5%), followed by 0.25 V (56 ± 5%) and −0.25 V (49 ± 6%). In contrast, higher electron distribution to CH_4_ was noticed at −0.25 V (41 ± 2%), followed by 0.25 V (29 ± 6%) and 0 V (23 ± 1%). CH_4_ was the largest electron sink (73 ± 4%) in the O.C reactors. In general, the electron distribution for undefined sinks was highest in O.C reactors, whereas in the SAP-MECs higher electron distribution to undefined sinks was noticed at 0.25 V (15.25 ± 0.9%) followed by −0.25 V (9.6 ± 2.8%) and 0 V (6.1 ± 2.7%).

### Experimental distribution of electrons to acetate and formate during propionate oxidation over the course of the last batch cycle

**Figure 2:**
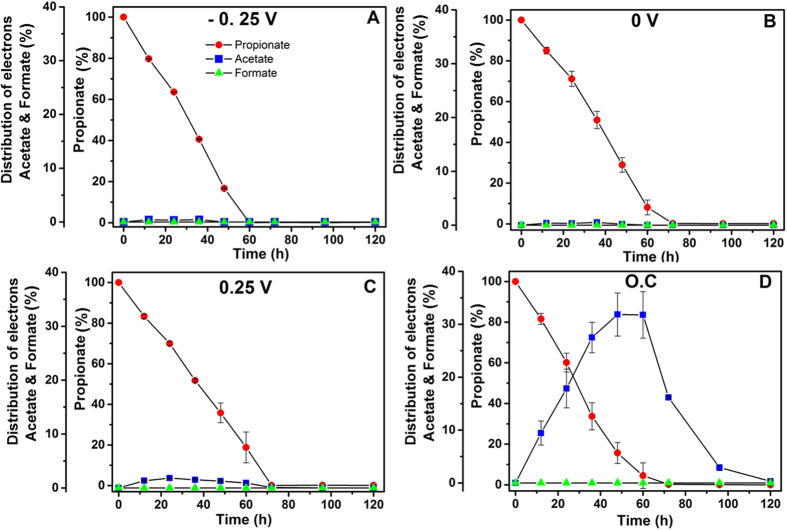
Distribution of electrons to acetate and formate during the oxidation of propionate in the anode over the course of a batch experiment in all SAP and O.C reactors. (**A**) −0.25 V SAP-MEC, (**B**) 0 V SAP-MEC, (**C**) 0.25 V SAP-MEC and (**D**) O.C reactors.

After 155 days of operation, the distribution of electrons to acetate and formate during the oxidation of propionate was examined over the course of a batch cycle (Fig. 2). The gas bag analysis adapted in this study does not allow analysis of H_2_ in multiple points in a single batch cycle. Propionate was completely consumed within ~60–70 hours in all the SAP-MECs and O.C reactors. The propionate removal rate was approximately similar in all the tested SAP-MECs (10.5 ± 0.4 mM/day) and O.C (9.02 ± 0.5 mM/day) reactors. However, accumulation of acetate (9.9 ± 0.2 mM; 15.85 ± 0.6% of electrons) was noticed in the O.C reactors at time ~70 hours of the batch, and it gradually decreased to reach 0% at time 120 hours. No accumulation or minor concentrations of acetate (<0.4 mM) were measured for the SAP-MECs (Fig. 2). Formate was below the detection limit throughout the batch of the SAP-MEC and O.C reactors (Fig. 2).

### Cyclic Voltammetry

**Figure 3:**
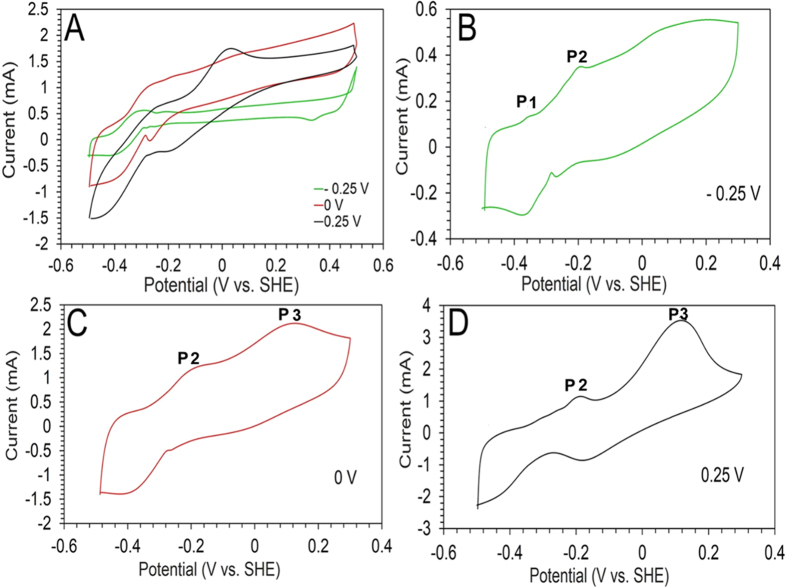
Cyclic voltammograms (CVs) of the biofilms (155 days) enriched at different SAP (−0.25, 0, and 0.25 V vs. SHE). (**A**) CV performed at the start of the batch in fresh growth medium with sodium propionate (36 mM) with a scan rate of 1 mV/s. (**B**–**D**) CV performed at the end of the batch with depleted sodium propionate growth medium with a scan rate of 1 mV/s.

CV analysis was performed for the biofilms (155 days aged) enriched at different SAPs immediately after feeding with a fresh growth medium (pH 8.9; 36 mM sodium propionate) to examine the influence of imposed anode potential on biofilm redox behavior. All biofilm voltammograms showed catalytic behavior with a rise in oxidation current between the potential window of −0.4 to −0.3 V (Fig. 3A). Nonturnover CV analysis of the respective biofilms in propionate limited growth medium exhibited three distinct oxidation peaks at, −0.34 V (P1), −0.19 V (P2), and 0.11 V (P3) (Fig. 3B–D). The CV analysis of the virgin anode in propionate growth medium (cell free) and reactor effluent (filtered and unfiltered) did not demonstrate any redox peaks (data not shown). Thus, the observed redox peaks from biofilm-voltammetry were presumably from the anodic biofilm and/or bacterial self-induced mediators (if localized in the biofilm matrix).

### Analysis of microbial community

A total of 804,695 high-quality reads (average length of ~450 bp) were generated after denoising, quality filtering and removal of chimeric sequences. For alpha diversity measures, the dataset (normalized abundance values) was subsampled to an even depth of 44,170 sequences across the samples to remove inherent heterogeneity of sampling depth. This number was chosen, as it corresponds to the lowest number of sequence reads detected. The diversity values across the anode samples ranged as follows: observed operational taxonomic units (OTUs: 1,006–1,679), Chao 1 (3,148–5,551), Shannon diversity index (H; 3.3–5.4), Simpson diversity index (D; 0.69–0.92) and phylogenetic diversity (PD; 65–91) (Supplementary Table S2). The highest diversity based on observed OTUs, Chao 1, H, D and PD was observed in the anode of O.C reactors. Whereas, the lowest diversity based on Chao 1, H, D and PD was observed in the SAP-MEC operated at 0 V (Supplementary Table S2). All five indices, that is, observed OTU, Chao 1, H, D and PD, demonstrated that the suspension samples have higher diversity than the anode samples among the four reactor types (i.e. −0.25 V, 0 V, 0.25 V and O.C). Good’s coverage (90–94%) revealed that the 16S rRNA gene sequences identified in these samples represent the majority of bacterial diversity present in each sample. A Venn diagram showing the shared OTUs among the three anodes (−0.25, 0 and 0.25 V) was prepared by normalizing the sequence reads to 57,043, which was lowest number of sequence reads detected for the three anodes (Supplementary Figure S3). Of the 9,400 total observed OTUs, only 580 OTUs (6.2%), 760 OTUs (8.1%), 932 OTUs (9.9%) and 819 OTUs (8.7%) were shared between the three anodes (−0.25, 0 and 0.25 V), between −0.25 and 0 V, between 0 and 0.25 V, and between 0.25 and −0.25 V (Supplementary Figure S3). On the other hand, the unique OTUs for the −0.25, 0 and 0.25 V correspond to 27.1% (2545 OTUs), 26.5% (2491 OTUs) and 32.1% (3013 OTUs) of the total observed OTUs (Supplementary Figure S3).

**Figure 4:**
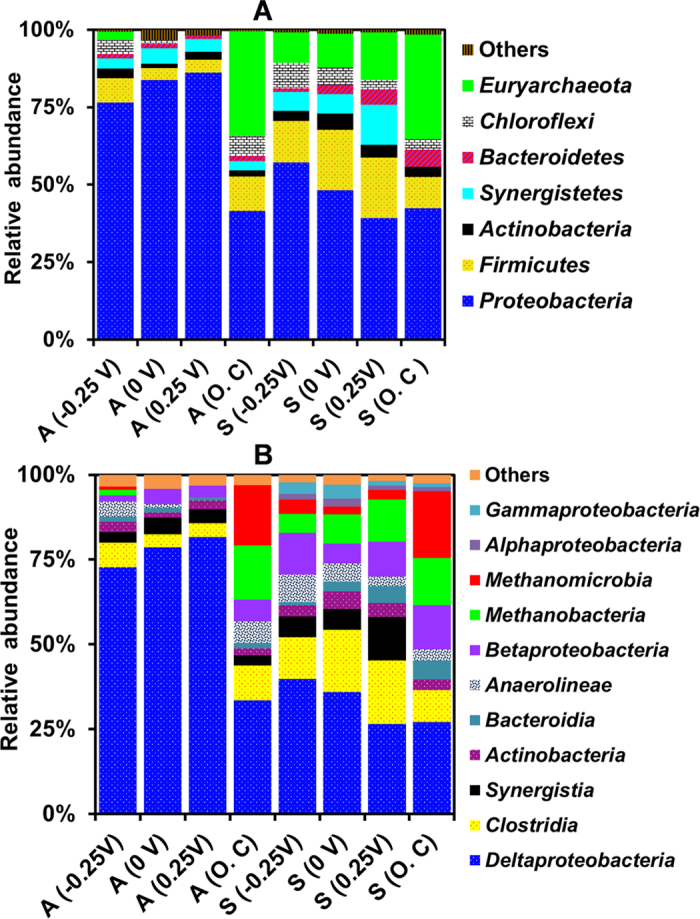
Relative abundance of the microbial communities that developed at (**A**) phylum and (**B**) class level for the different SAP (−0.25, 0, and 0.25 V vs. SHE) and O.C reactors. “A” and “S” correspond to the anode and suspension samples. Microbial communities representing less than 1% of the total sequence reads are classified as others.

The phylum level classification of the anodes enriched at different SAPs and O.C conditions showed the dominance of *Proteobacteria* by 82 ± 5% and 41 ± 3%, respectively (Fig. 4A). Additionally, *Firmicutes, Synergistetes, Actinobacteria* and *Bacteroidetes* were detected in all the anodes of SAP-MECs, but to a lesser extent than *Proteobacteria* (Fig. 4A)*. Chloroflexi* and *Euryarchaeota* were present only in the anode of −0.25 V. In addition, *Euryarchaeota* was highly abundant in the anode of O.C Fig. 4A). Likewise, the suspension samples of all the reactors were dominated by *Proteobacteria, Firmicutes, Euryarchaeota* and *Synergistetes,* and to a lesser extent by *Chloroflexi, Actinobacteria* and *Bacteroidetes* (Fig. 4A).

The class level classification of the anodes poised at different SAPs indicated the predominance of *Deltaproteobacteria* (78 ± 5%) and it was significantly lower in the anode of O.C (33 ± 2%) as well as in the suspension (32 ± 6%) of all the samples (*p* < 0.05) (Fig. 4B). All the anodes and suspension samples contained *Clostridia, Synergistia, Actinobacteria, Betaproteobacteria* and *Bacteroidia* (Fig. 4B)*. Anaerolineae* was present in all the samples (anode and suspension) except the anode of 0.25 V (Fig. 4B). The methanogenic classes *Methanomicrobia* and *Methanobacteria* were only present in the anode of −0.25 V, but as a minor fraction of the total community. However, they were highly abundant in the anode of O.C and in all the suspension samples (Fig. 4B).

**Figure 5:**
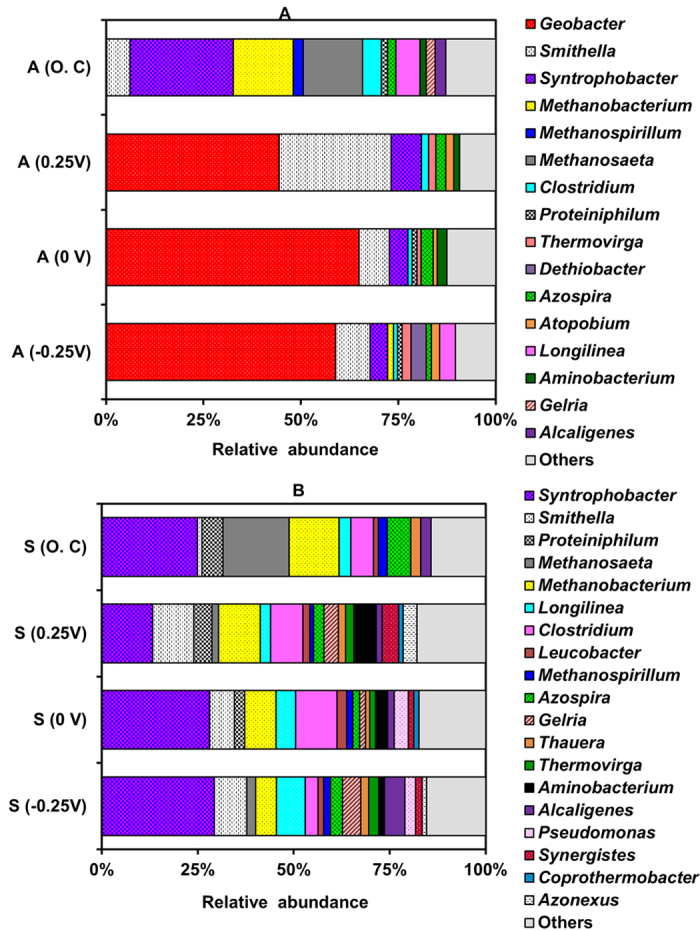
Relative abundance of the microbial communities at the genus level for the different SAP (−0.25, 0, and 0.25 V vs. SHE) and O.C reactors. (**A**) anode samples and (**B**) suspension samples. “A” and “S” correspond to the anode and suspension samples. Microbial communities representing less than 1% of the total sequences reads are classified as others.

At the genus level, all the anodes of SAP-MECs were dominated by *Geobacter* with sequences most similar to *Geobacter sulfurreducens* (99.5% similarity). Notably, *Geobacter* was highly abundant in the anode of 0 V (65 ± 5%), followed by −0.25 V (59 ± 3%) and significantly lower in the anode of 0.25 V (45 ± 2.6%) (*P* < 0.05) (Fig. 5A). The anode of O.C showed abscence of *Geobacter.* Moreover, *Smithella* was abundant in the anode of −0.25, 0 V and O.C (8 ± 1.4%) and it was remarkably more abundant in the anode of 0.25 V (29 ± 3%). *Syntrophobacter* with sequences most similar to *S. sulfatereducens* (99.4% similarity) was observed in all the anodes of SAP-MECs (6 ± 2%) and it was significantly higher in the anode of O.C (27 ± 5%) (Fig. 5A). Additionally, *Methanobacterium* most similar to *M. formicicum* (99.5% similarity) was detected in the anode of −0.25 V (1.5 ± 0.3%) and it was present at a higher abundance in the anode of O.C (15.4 ± 2.5%). Other methanogens detected in the anode of O.C were *Methanosaeta* (15 ± 2%) most similar to *M. concilii* (99.5% similarity) and *Methanospirillum* (2.5 ± 0.6%), both belonging to the class *Methanomicrobia* (Fig. 5A).

The suspension samples of SAP-MECs and O.C reactors were highly diverse and dominated by a wide range of microorganisms (Fig. 5B). *Syntrophobacter* was highly abundant in all the suspension samples (24 ± 7%). Likewise, *Smithella* was abundant in all the suspensions of SAP-MECs (9 ± 2%), but was present in lower abundance in the suspension of O.C (1.2 ± 0.3%). Additionally, all the suspension samples contain high abundance of *Methanobacterium* (9.4 ± 3.3%). *Methanosaeta* was present in low abundance (2 ± 1%) in the suspensions of SAP-MECs, but was highly abundant (17 ± 3%) in the suspension of O.C (Fig. 5B).

The microbial composition of the inoculum at the phylum level was dominated by *Proteobacteria* (68%), *Firmicutes* (13.5%), *Chloroflexi* (6%), *Bacteroidetes* (6%), and *Euryarchaeota* (1%) (Supplementary Figure S4A). The dominant genera detected in the inoculum were *Pseudomonas, Gelria, Proteiniphilum, Longilinea, Azospira, Smithella, and Methanosaeta* (Supplementary Figure S4B).

**Figure 6:**
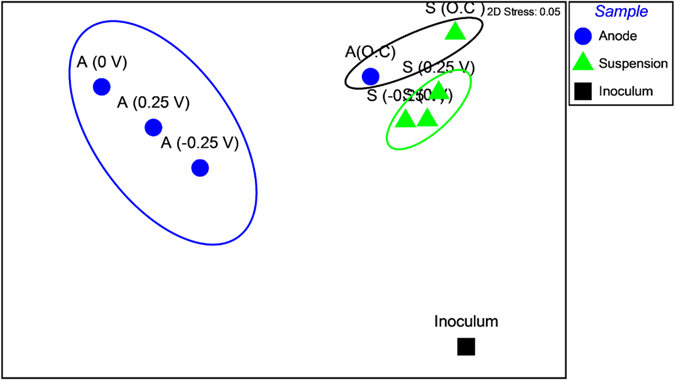
Nonmetric multidimensional scaling plot derived from unweighed Unifrac distance for the different SAP-MEC (−0.25, 0, and 0.25 V vs. SHE) and O.C reactors. “A” and “S” correspond to the anode and suspension samples.

The ratio of Archaea to Bacteria was significantly higher in the anode and suspension of O.C than the anode and suspension of SAP-MECs (Supplementary Figure S5). Also, the ratio was higher in the suspension than the anode samples of SAP-MECs. Non-metric multidimensional scaling (NMDS) showed that the anode and suspension communities of SAP-MECs differed substantially from the original inoculum source (Fig. 6). Three clear and distinct clusters can be obsereved in the NMDS plot. The three custers correspond to the anode communities in the SAP-MECs, the suspension communities in the SAP-MECs and the anode and suspension communities in the O.C reactors (Fig. 6).

## Discussion

The results gathered in this study show that SAPs influenced MEC performance as supported by differences in the electron distribution to various sinks (Table 1). Electrical current was the largest electron sink regardless of the tested SAPs (Table 1) and it was relatively higher in the positive SAPs (0 and 0.25 V) than negative SAP (−0.25 V). In general, at positive SAPs, exoelectrogens can gain more energy for growth and increase their metabolic rate^31^. At elevated metabolic rates, more substrate is consumed by exoelectrogens for current production and less substrate is available for other competing reactions like methanogenesis (Table 1)^31^. In addition, based on thermodynamics alone (see Supporting Information), and under standard biochemical conditions (i.e., reactants and products at 1 M or 1 atm, 298 K and pH 7), oxidation of acetate to electrical current is more favorable at the positive SAPs tested in this study (0 V: 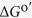 = −214.6 kJ/mol and 0.25 V: 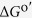 = −407.6 kJ/mol) than the negative SAP (−0.25 V: 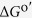 = −21.6 kJ/mol). Similarly, oxidation of H_2_/formate to current is more favourable at postive SAPs (0 V: 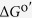 = −79.7–94.4 kJ/mol and 0.25 V: 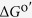 = −128–142.6 kJ/mol) than negative SAP (−0.25 V: 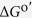 = −31.5–46.1 kJ/mol). The electron distribution to electrical current at 0 (71%) and 0.25 V (56%) (Table 1) was higher than the theoretical electron distribution to current from acetate oxidation alone (48%) (see Supplementary Information), suggesting that both acetate and H_2_/formate contributed to current generation at these SAPs. Furthermore, at 0.25 V, the current generation from intermediates (acetate: 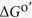 = −407.6 kJ/mol, H_2_: 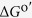 = −128 kJ/mol, and formate: 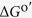 = −142.6 kJ/mol) is thermodynamically more favorable than 0 V (acetate: 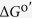 = −407.6 kJ/mol, H_2_: 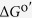 = −214.6 kJ/mol, and formate: 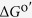 = −94.4 kJ/mol) (see Supporting Information). However, results showed that electrical current was a larger electron sink at 0 V than 0.25 V (Table 1). Perhaps the additional energy produced at 0.25 V could not be captured by exoelectrogens, and instead it was captured by other microorganisms, which resulted in significant losses to undefined sinks (~2.5 times higher than 0 V) and methane (Table 1) (P < 0.05).

Methane production occurred at all SAPs and it corresponded to the second largest electron sink (23–41%) (Table 1). Most MEC studies showed that methane generation was mainly due to hydrogenotrophic methanogens because acetate-oxidizing exoelectrogens (e.g. *G. sulfurreducens*) could outcompete acetoclastic methanogens for acetate because of kinetic benefits^16^,^18^,^32^. Acetoclastic methanogens were not detected at a SAP of 0 V, and were present at very low abundance (1.7 ± 0.2%) at a SAP of 0.25 V, suggesting that the methane generated was due to hydrogenotrophic methanogens (Fig. 5). Interestingly, the MECs operated at SAPs of −0.25 V exhibited a higher fraction of electrons distributed to CH_4_ (41 ± 2%) (Table 1) than the theoretical electron distribution to CH_4_ via hydrogenotrophic methanogenesis (36%) (see Supplementary Information and Supplementary Figure S6), suggesting that methane generation could have happened through hydrogenotrophic (H_2_/formate pathways) and acetoclastic methanogenesis (acetate pathway). Indeed, both hydrogenotrophic (*M. formicicum* and *Methanospirillum*) and acetoclastic methanogens (*M. concilli*) were detected in the suspension of −0.25 V (Fig. 5B). Also, based on thermodynamics alone (see Supplementary Information), and under standard biochemical conditions, oxidation of acetate to CH_4_ (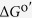 = −31 kJ/mol)^33^ is preferable than the direct oxidation of acetate to electrical current (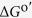 = −21.6 kJ/mol). Hence, at more negative SAP (−0.25 V), acetate also likley contributed for CH_4_ production. Similarly, at SAP of −0.25 V, oxidation of H_2_/formate to CH_4_ (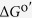 = −136 and −130 kJ/mol) is thermodynamically more favorable than oxidation of H_2_/formate to electrical current (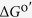 = −31.5 and −46.1 kJ/mol). Therefore, at more negative SAP (−0.25 V), a major fraction of electrons were lost to CH_4_ (Table 1). As expected, O.C reactors showed a higher fraction of electrons routed for CH_4_ (73 ± 4) via acetoclastic and hydrogenotrophic methanogens, which were both abundant in the anode and suspension (33 ± 0.4%) (Fig. 5).

The results revealed that SAPs in propionate-fed MECs affected the microbial community structure of the anode. The most dominant genera detected on the anode of all three SAPs were *Geobacter, Smithella* and *Syntrophobacter*, but their relative abundance varied among the tested SAPs (Fig. 5A). Also, more positive SAP (0.25 V) showed higher microbial diversity than 0 V and −0.25 V as indicated by higher observed OTUs, Chao 1 and PD (Supplementary Table S2). In an acetate-fed MEC, Torres *et al*. observed the highest phylogenetic diversity at positive SAP (0.37 V vs. SHE) compared to other tested SAPs (−0.15, 0.09 and 0.02 V vs. SHE), which were mainly dominanted by *G. sulfurreducens* (90%)^11^. It is likely that the additional energy gain produced at 0.25 V in the current study was not fully captured by *Geobacter,* and instead it was captured by other diverse microorganisms that interfered with *Geobacter* to make direct contact with the anode^11^. This explains why *Geobacter* was much lower in the anode set at 0.25 V (45%) compared to 0 V (65%) and −0.25 V (59%).

*Geobacter* (99.5% similarity to *G. sulfurreducens*) was the dominant genera detected in the anode of the three SAPs tested in this study (average percentage of 56 ± 10%). Previous studies also revealed the dominance of *G. sulfurreducens* or different species/strains of *Geobacter* phylotypes at varying SAPs in MXCs fed with acetate^1^,^6^,^12^,^14^. According to previous studies, *G. sulfurreducens* can self-adjust their electron transfer pathways (ETP) to adapt to different SAP conditions^1^,^34^,^35^. Zacharood *et al*. showed that two different types of c-type cytochromes (ImcH and CbcL) were involved in ETP at lower and higher SAPs^36^. Based on nonturnover CVs, three oxidation peaks (P1, P2 and P3) were observed in the current study (Fig. 3B–D). The redox couple at ~−0.2 V (P2 position in Fig. 3) has been observed in previous studies^1^,^37^,^38^, and it is similar to the midpoint potential of the redox couple expressed in *G. sulfurreducens* matured biofilms for turnover of electrons to the anode^38^. Also this potential (−0.2 V) is close to the midpoint potential of periplasmic cytochrome C (PpcA, −0.17 V), multiheme cytochrome OmcB (−0.19 V), and OmcZ (−0.22 V)^38^,^39^,^40^. Peak P1 (−0.34 V) matches previously reported CV of *G. sulfurreducens* biofilm in the potential range of −0.32 V^37^.

Although *G. sulfurreducens* was detected in high abundance at the anodic biofilm, the profile of the voltammograms recorded in this study did not follow the typical shape of *G. sulfurreducens* voltammograms recorded in a previous MEC study using acetate as the fuel substrate and graphite fiber brush as the anode^41^. Presumably the fermentable nature of the substrate used in this study, which requires syntrophic cooperation between fermenters and exoelectrogens for its degradation, affected the voltammogram measurements in this study.

Other dominant genera in the anode were related to *Smithella* and *Syntrophobacter* (99.4% similarity to *S. sulfatereducens*). These bacteria act as the main functional groups responsible for syntrophic propionate degradation in methanogenic bioreactors fed with propionate as the sole carbon source^42^,^43^,^44^. Their presence at the anode suggests a syntrophic interaction with *Geobacter* for propionate oxidation. *Smithella* and *Syntrophobacter* were also present at relatively high abundance in the suspension of SAPs along with hydrogenotrophic methanogens. Notably, the relative abundance of *Smithella* was three times higher in the anode of more positive SAP-MECs (0.25 V) than 0 and −0.25 V, and at the moment there is no explanation for this observation. (Fig. 5A). Recently, a fermentative bacterium (*Thermoanaerobacter pseudethanolicus*) was shown to have the capability to simultaneously ferment sugars (xylose, glucose and cellobiose) and convert fermentation product (acetate) to current in MECs operated at fixed anode potential (0.042 V vs SHE)^45^. Therefore, it is tempting to test if other fermentative organisms like *Smithella* could utilize the anode through acetate during fermentation of propionate when the potential becomes favourable. Thus, future research is needed to provide a deeper insight of the role of *Smithella* at various SAP conditions in MECs fed with propionate.

In this study, complete degradation of propionate (Table 1) was observed in all the tested conditions (SAPs and O.C). No accumulation of its intermediates (acetate and formate/H_2_) (<0.4 mM) was observed throughout the batch in all SAP-MECs (Fig. 2). However, accumulation of acetate was noticed in the O.C reactors (Fig. 2D). In SAP-MECs, both *Geobacter* and hydrogenotrophic methanogens consumed the intermediates generated by fermenters (*Smithella* and *Syntrophobacter*) (Fig. 5), and kept their concentrations low resulting in more energetically favourable fermentation, and hence complete removal of propionate. In the O.C reactors, complete removal of propionate was facilitated through microbial partnerships between fermenters (*Smithella* and *Syntrophobacter*) and hydrogenotrophic and acetoclastic methanogens. In our earlier study^30^, we demonstrated that propionate degradation in MECs occurred via a microbial partnership between fermenters and *Geobacter*, and low propionate removal (80% removal) was observed at 36 mM (similar to the concentration used in the current study) compared to complete removal at 4.5 mM^30^. Methanogenesis was not an important electron sink in our earlier study at both propionate concenrations tested, whereas methanogenesis (via hydrogenotrophic methanogens) was an important sink in the current sudy. In our previous study^30^, MEC anodes were initially enriched (30 days) in single chamber air-cathode MFCs, where oxygen intrusion through the cathode might have affected the growth of methanogens. Whereas in the current study, the reactors were operated under MEC (anaerobic) mode from the start of the experiment, and this provided a suitable environment for the enrichment of hydrogenotrophic methanogens. Based on the aforementioned studies, it seems that the complete degradation of propionate at elevated concentrations in MECs requires microbial partnerships between fermenters, hydrogenotrophic methanogens and *Geobacter*. Whereas, at low propionate concentration microbial partnership between *Geobacter* and fermenters was sufficient to achieve complete removal of propionate in MEC^30^.

## Conclusion

This study is the first to report the effect of SAPs on propionate-fed MECs. The resutls showed that (i) SAPs affected the electron fluxes to various electron sinks, where current was a significant electrons sink followed by methane in all the SAPs tested; however, current was relatively higher in the positive SAPs (0 and 0.25 V). In contrast, methane was higher in the negative SAP (−0.25 V); and (ii) SAPs affected the anodic microbial community struture and diversity where higher microbial diversity was detected at SAP of 0.25 V than SAP of 0 and −0.25 V, and the relative abundance of the most dominant members (*Geobacter, Smithella* and *Syntrophobacter*) at the anode varied among the tested SAPs. A complete oxidation of propionate was observed with no accumulation of its intermediates in all SAP-MECs. Microbial community composition in the biofilm anode and suspension imply that the degradation of propionate in all the tested SAPs was fascilitated by syntrophic interactions between fermenters (*Smithella* and *Syntrophobacter*) and *Geobacter* in the anode and fermenters (*Smithella* and *Syntrophobacter*) and methanogens (mainly hydrogeonotrophic methanogens) in suspension.

## Methods

### Construction and operation of MECs

Two-chambered, cube-shaped MECs with a working volume of 40 mL (anode chamber) and 20 mL (cathode chamber) were constructed as previously described^30^. The two chambers were separated by an anion exchange membrane (5 cm^2^; AMI 7001, Membranes International, Glen Rock, NJ). A glass gas collection tube (15 mL) was attached to the top of both the anode and cathode chambers. Gas bags (0.1 L Cali-5-Bond. Calibrate, Inc.) were connected to the top of the glass collection tubes to collect more volume of gas. The anodes were graphite fiber brushes (2.5 cm diameter × 2 cm long; PANEX 33 fibers, ZOLTEK Inc., St. Louis, MO, USA). The cathodes (projected surface area of 7 cm^2^) were made using carbon cloth (type B-1B, E-TEK) containing 0.5 mg/cm^2^ of Pt on the side facing the anode^46^, and four polytetrafluoroethylene diffusion layers on other side^30^. An Ag/AgCl reference electrode (+210 mV vs SHE; RE-5B; BASi) was placed between the electrodes. Necessary precautions were taken for positioning the working, counter and reference electrodes in the reactor as recommended by Zhang *et al*.^41^, to provide uniform potential distribution across the working electrode^41^. All potentials were reported here versus SHE for comparison to other studies.

All MEC anodes were inoculated with effluent from anaerobic membrane bioreactor that had been operated with synthetic municipal wastewater medium (containing starch, milk powder and yeast) for more than one year in our lab. The anaerobic membrane bioreactor was inoculated with camel manure (Jeddah, KSA) and anaerobic digested sludge (Manfouha Wastewater Treatment Plant, Riyadh, KSA). The anode and cathode medium (pH 8.9) consisted of bicarbonate buffer (80 mM NaH_2_CO_3_), nutrients (6 mM NH_4_Cl, 0.4 mM Na_2_HPO_4_, 0.2 mM NaH_2_PO_4_), Wolfe’s vitamin (10 mL/L) and trace mineral (10 mL/L) solutions^30^,^47^,^48^. The anode medium was supplemented with propionate (36 mM) as the sole carbon and energy source. The medium was boiled and then cooled to room temperature by sparging with N_2_:CO_2_ (80:20, vol/vol) gas mix for 30 min to remove any dissolved oxygen and was then autoclaved. The MECs were operated with three different SAPs (−0.25, 0 and 0.25 V) using a potentiostat (VMP3; Biologic, Claix, France) in a temperature controlled room (30 °C), with data recorded at 10-min intervals and analyzed using EC-lab V10.02 software. The control MECs were operated in an open circuit (O.C) mode. All reactor types were run in duplicate and operated in a fed-batch mode. When the current dropped to almost 0 mA (~4–5 days/cycle), the reactors were refilled with fresh medium and sparged with nitrogen gas (99.999%).

### Electron balance

To establish electron balances during the time course or at the end of a batch cycle, the distribution of electrons in milli e^−^ equivalents (m e^−^eq) in the anode of MECs was followed from the electron donor (propionate) to various measured electron sinks (current, H_2_, CH_4_, propionate, acetate, and formate). The concentrations of propionate, formate, and acetate were analyzed by high-performance liquid chromatograph (HPLC) (Thermo Scientific, Accela, country). Samples for HPLC analysis were collected during the time course or at the end of the batch. The concentrations of H_2_ and CH_4_ were measured using a gas chromatograph (GC) (model 310; SRI Instruments). Samples for GC analysis were collected at the end of the batch. The details of GC and HPLC analysis are provided in the Supplementary Information.

### Calculations

Hydrogen production rate at the cathode, *Q* (m^3^ H_2_/m^3^ reactor/day); volumetric current density of the reactor, *I*_V_ (A/m^3^); hydrogen yield, *Y*_H2_ (mol-H_2_/mol-propionate consumed); coulombic efficiency, CE (%); propionate removal (%) were determined as previously described^30^. CE is defined as the fraction of the electrons recovered as electrical current compared to the theoretical electrons obtained from the complete oxidation of the substrate (i.e. propionate)^30^.

### Electroanalysis

Cyclic Voltammetry (CV) was performed immediately after feeding with fresh growth medium containing sodium propionate (36 mM). Scans ranged from −0.5 to 0.5 V (turnover) and −0.5 to 0.3 V (non-turn over) at a rate of 1 mV/s, with the anode as the working electrode, the cathode as the counter electrode and Ag/AgCl as the reference electrode. Nonturnover CVs were performed when the current in MECs decreased to almost 0 mA with depleted sodium propionate growth medium. Electrochemical impedance spectroscopy analysis was carried out to measure the working electrode (i.e. carbon fiber brush anode) ohmic resistance at different set potentials^49^. Two-chambered, cube-shaped MEC was used for the measurements with cell-free growth medium as the electrolyte. The ohmic resistance of the anode determined from Nyquist plot was used to measure the ohmic drop compensation.

### 16S rRNA gene sequencing

At the end of the experiments (day 155), samples were collected from the anode and suspension of SAP-MECs and O.C reactors for microbial community analysis. The details of genomic DNA extraction, PCR, sequencing and data analysis are provided in the Supplementary Information.

### Nucleotide sequence accession numbers

The 16S r RNA gene sequencing reads have been deposited in European Nucleotide Archive under the accession number PRJEB14918.

## Additional Information

**How to cite this article**: Hari, A. R. *et al*. Set anode potentials affect the electron fluxes and microbial community structure in propionate-fed microbial electrolysis cells. *Sci. Rep.* **6**, 38690; doi: 10.1038/srep38690 (2016).

**Publisher's note:** Springer Nature remains neutral with regard to jurisdictional claims in published maps and institutional affiliations.

## Supplementary Material

Supplementary Information

## Figures and Tables

**Table 1:** Distribution of electrons in the anode at the end of the last batch cycle for all SAP and O.C reactors.

Electron sinks	Fraction of electrons at the end of batch tests (%)^a^			
	−0.25 V	0 V	0.25 V	O.C
Electrical current	49 ± 6	71 ± 5	56 ± 5	0
Methane	41.1 ± 2	22.9 ± 1.2	28.7 ± 6.3	73 ± 4
Hydrogen	0.04 ± 0.01	0.03 ± 0.01	0.05 ± 0.02	0.07 ± 0.01
Propionate	b.d.l^b^	b.d.l	b.d.l	b.d.l
Acetate	b.d.l	b.d.l	b.d.l	b.d.l
Formate	b.d.l	b.d.l	b.d.l	b.d.l
Undefined sinks^c^ (biomass + soluble microbial products + others)	9.6 ± 2.8	6.1 ± 2.7	15.25 ± 0.9	27.03 ± 2.1

The crossover of propionate and its intermediates to the cathode through the anion exchange membrane was minimal (<0.5%). Therefore, they were not included in the calclulations.

^a^Values represent the average of the duplicate reactors.

^b^b.d.l below detection limit.

^c^e^−^ (undefined electron sinks) = e^−^ (propionate)–e^−^ (current)–e^−^ (unused propionate)–e^−^ (acetate)–e^−^ (formate)–e^−^ (hydrogen)–e^−^ (methane), where e^−^ = electron equivalents.
