# Supplementary material for: Set anode potentials affect the electron fluxes and microbial community structure in propionate-fed microbial electrolysis cells
Source: Sci Rep. 2016 Dec 9;6:38690. doi: 10.1038/srep38690 (PMC5146674; doi:10.1038/srep38690)
Supplement: Supplementary Information [file srep38690-s1.pdf]

Set anode potentials affect the electron fluxes and microbial community structure in propionate-fed microbial electrolysis cells

Ananda Rao Hari[1], Krishna P. Katuri[1], Bruce E. Logan[2], and Pascal E. Saikaly[1,*]

[1]King Abdullah University of Science and Technology, Biological and Environmental Sciences and Engineering Division, Water Desalination and Reuse Research Center, Thuwal 23955-6900, Saudi Arabia
[2]The Pennsylvania State University, Department of Civil and Environmental Engineering, University Park, PA 16802, USA

*Corresponding author: Pascal Saikaly (pascal.saikaly@kaust.edu.sa)

Supplementary Methods

Analyses. The gas generated by the MECs was collected from both chambers in the head space and in a gas bag (0.1 L Cali -5 -Bond. Calibrate, Inc.), and the total gas volume was measured by the gas bag method as previously described [1]. Concentrations of H_2, N_2, CH_4 were measured using a gas chromatograph (GC) (model 310; SRI Instruments) with a thermal conductivity detector (TCD), a 1.83-m molecular sieve packed 5A column, and an argon carrier gas. CO_2 concentration was measured with a second GC (model 310; SRI Instruments) with a TCD, a 0.91-m silica gel column, and using helium as the carrier gas. The volatile fatty acids (VFAs) profile (propionate, formate, and acetate) and their concentrations were analyzed by high-performance liquid chromatograph (HPLC) (Thermo Scientific, Accela, country) equipped with a photo-diode array (210 nm) and an ultraviolet detector. An Aminex HPX-87H column (Bio-Rad Laboratories, Hercules, CA, USA) was used to separate the VFAs. Sulfuric acid (5 mM) was used as the mobile phase at a flow rate of 550 µL/min, and the pressure was maintained at 9650 kPa. The total elution time was 30 min, each sample was measured in triplicate, and the average concentrations were reported [2].

16S rRNA gene sequencing. Genomic DNA was extracted using the PowerBiofilm DNA extraction kit (MO BIO Laboratories, Inc., Carlsbad, CA) following the manufacturer's instructions. The quality (A260/A280) and quantity (A260) of the extracted genomic DNA was determined using a Nanodrop 1000 spectrophotometer (Thermo Fisher Scientific, Waltham, MA).

Triplicate PCR reactions were performed for each sample in a 25 µL reaction volume using the HotStarTaqPlus Master Mix (Qiagen, Valencia, CA), 0.5 µM of each primer, and 100-200 ng

of template DNA. The V3-V4 hypervariable region of 16S rRNA genes were amplified using a universal primer set for prokaryotes: Pro 341F (5'-Illumina adapter-Barcode-Linker-CCTACGGGNBGCASCAG-3') and Pro 805R (5'-5'-Illumina adapter-Linker -GACTACNVGGGTATCTAATCC-3') [3]. PCR was performed using life technologies veritus thermocycler with the following PCR conditions: initial denaturation at 94°C for 3 min, followed by 28 cycles of denaturation at 94° C for 30 seconds, annealing at 53° C for 40 seconds, extension at 72° C for 1 min and a final extension at 72° C for 5 min.

Following PCR, all amplicon products from the different samples were mixed in equal concentrations, purified using Agencourt Ampure beads (Agencourt Bioscience Corporation, MA, USA), and sequenced on the Illumina TruSeq technology (San Diego, CA) according to manufacturer's instructions.

The 16S rRNA sequences were processed using the Quantitative Insights Into Microbial Ecology (QIIME v 1.9.0) pipeline [4]. Raw reads were first demultiplexed, trimmed and filtered for quality. The minimum acceptable length was set to 200 bp [4]. Sequences were clustered into operational taxonomic units (OTUs) at 97% sequence similarity using the uclust algorithm [5]. A representative sequence from each OTU was aligned using PyNAST [6], and these were phylogenetically assigned to a taxonomic identity (phylum, class and genus level) using the RDP Naive Bayesian rRNA classifier at a confidence threshold of 80% [7]. Chimeric sequences were identified and removed from the aligned sequences using chimera Slayer as implemented in QIIME. Rarified OTU tables were used to generate alpha and beta diversity metrics by normalizing to the lowest sequence read of the samples. For alpha diversity measurements, both non-phylogenetic based metrics (observed OTUs, Shannon diversity index (H), Simpson diversity index (D) and Chao 1 richness estimator) and phylogenetic based metric (phylogenetic

diversity (PD_whole)) were calculated with QIIME at the 3% distance level. Beta diversity metrics using the unweighted UniFrac distance matrix [8] was calculated and visualized with nonmetric multidimensional scaling (NMDS) using statistical software PRIMER 6 (version 6.1.13).

Table S1 comparative overview of the effect of SAPs in MEC

SAPs (V *vs*.SHE)	MEC configuration	Substrate	Inoculum	Working electrode	Electron fluxes	Microbial communities	References
-0.15, -0. 09, 0.02, 0.37	Dual chamber	Acetate (Non-fermentable)	Anaerobic digester sludge	Graphite rod	SAPs of -0.15 V, -0.09 V showed higher electron flux to current than the other tested potentials	Biofilms enriched at lower SAPs (-0.15 V, -0. 09 V, 0.02 V) were dominated by *G. sulfurreducens*. Whereas, biofilm grown under higher SAP (0.37 V) was enriched by a diverse bacterial community	9
-0.15, -0.09, 0.02	Dual chamber	Acetate (Non-fermentable)	Mixture of soil and activated sludge	Graphite rod	All the potentials tested showed relatively similar electron flux to current	All the potentials were dominated by *Geobacter spp.* However, 0.02 V dominated by *G. lovely* and -0.09 and -0.15 V dominated by *G. sulfurreducens*	10
-0.25, -0.09, 0.21, 0.51, 0.81	Single chamber	Acetate (Non-fermentable)	Effluent of primary clarifier	Graphite plate	0.21 V, 0.51 V, -0.09 V showed higher electron flux to current than the other tested SAPs	*G. sulfurreducens* was dominant in all the tested potentials	11
-0.2, -0.15, -0.1, 0	Dual chamber	Acetate (Non-fermentable)	Activated sludge/ anaerobic sludge	Graphite rod	0 V SAP showed higher electron flux to current and higher acetate degradation rate followed by -0.1 V, -0.15 V and -0.2 V	No information on microbial community was reported in their study	12
0	Dual chamber	Ethanol (fermentable)	Anaerobic digester sludge	Graphite rod	Hypothesis was tested at one SAP (0 V). Electron flow to current was increased to 84% when methanogenesis was inhibited	Ethanol-fermenting bacteria (largely belonging to *Pelobacter*) dominated the biofilm, followed by a diverse community of exoelectrogens, and hydrogenotrophic methanogens or homo-acetogens (in case of methanogen inhibition)	13
-0.25, 0, 0.25	Dual chamber	Propionate (fermentable)	Anaerobic digester sludge	Graphite brush	SAPs of 0 V and 0. 25 V showed higher electron flux to current. SAP of -0.25 V showed higher electron flux to methane	*Geobatcer sp*., *Smithella sp*. and *Syntrophobatcer sp*. were dominant at all tested SAPs. SAP of 0.25 V showed higher microbial diversity than the other tested potentials	This study

Supplementary Results

Table S2 Number of observed operational taxonomic units (OTUs) and alpha diversity measures

Samples	Number of observed OTUs	Richness estimate (Chao1)	Shannon Diversity Index (H)	Simpson Diversity Index (D)	Phylogenetic diversity (PD)	Good's coverage
A (-0.25 V)	1312	3918	4.7	0.89	75	0.98
A (0 V)	1006	3148	3.3	0.69	65	0.98
A (0.25 V)	1344	4100	3.9	0.78	77	0.98
A (O.C)	1679	5551	5.4	0.92	91	0.97
S (-0.25 V)	2190	5306	6.0	0.92	112	0.97
S (0 V)	2484	8010	6.7	0.96	120	0.96
S (0.25 V)	2499	8085	6.8	0.96	130	0.96
S (O.C)	2711	8930	5.9	0.93	124	0.96
Inoculum	1890	6268	5.7	0.93	95	0.97

"A" corresponds to anode and "S" corresponds to suspension

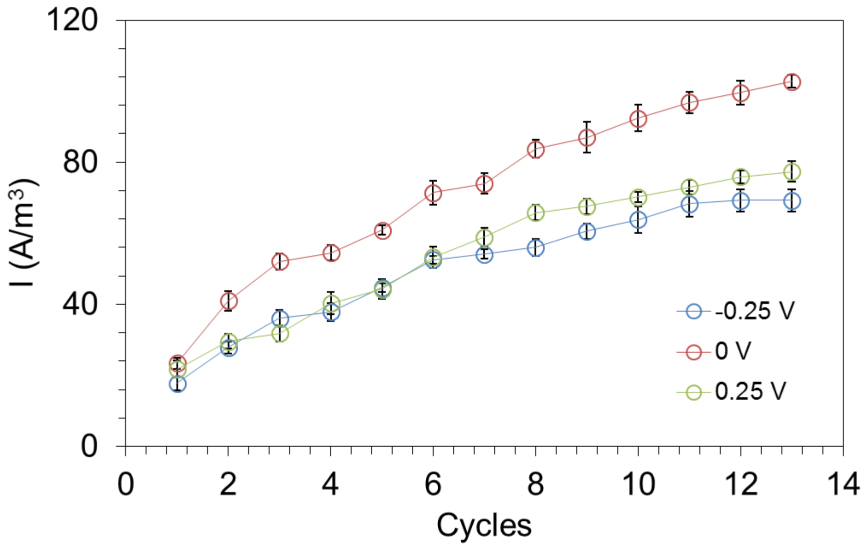

Figure S1 Maximum current density (average of duplicate MECs) profile for all the batch cycles of operation for reactors operated at different SAPs.

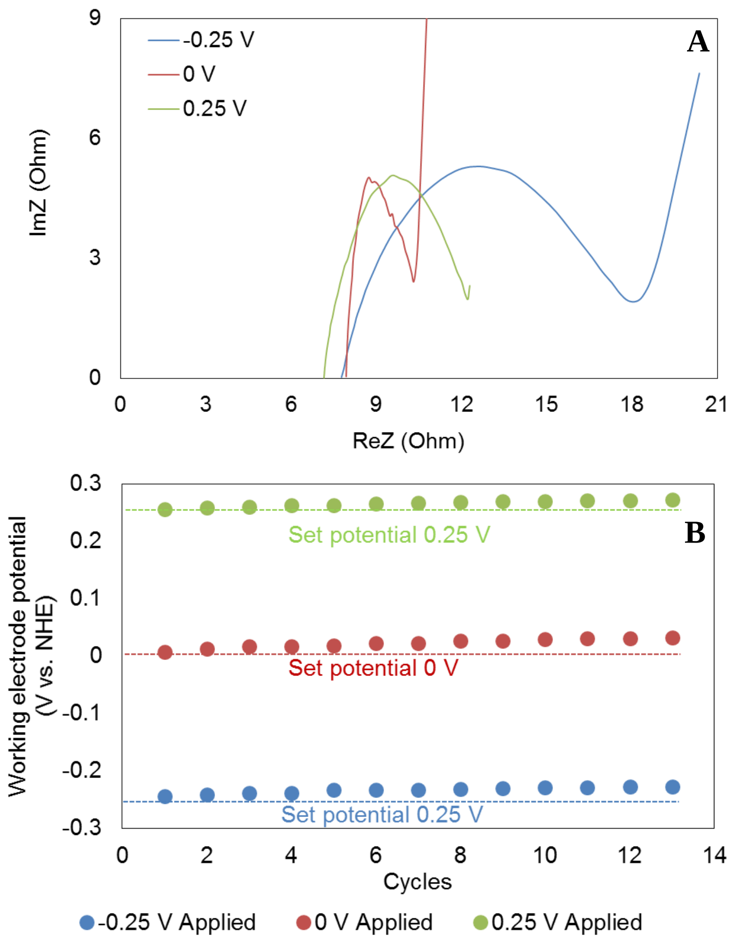

Figure S2 (A) Nyquist plots of electrochemical impedance spectroscopy spectra of the virgin carbon fiber brush anode performed at different set potentials in cell-free growth medium. (B) Profiles of measured potential of working electrode (circles; ohmic drop compensated) for all the batch cycles of MECs operated at different SAPs. The dashed lines correspond to the SAP of working electrode (uncompensated resistance).

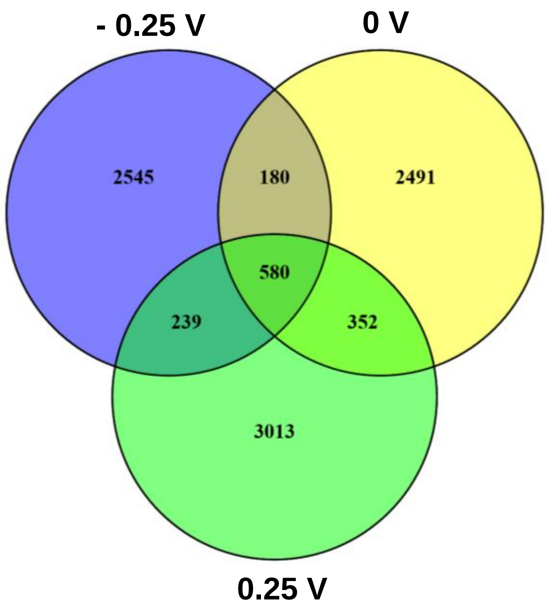

Figure S3 Venn diagram displaying shared and unique OTUs (3% distance cutoff) of the anode samples enriched at different SAPs.

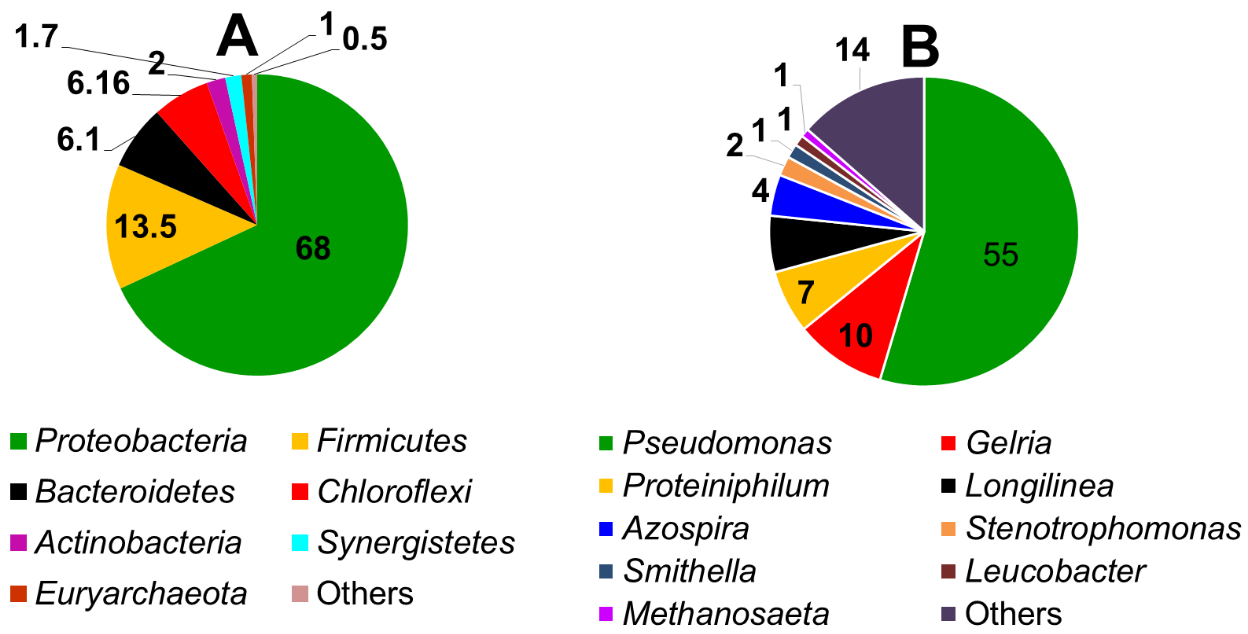

Figure S4 Relative abundance of the microbial communities of the inoculum (A) at phylum and (B) genus level. Microbial communities representing less than 1% of the total sequence reads are classified as others.

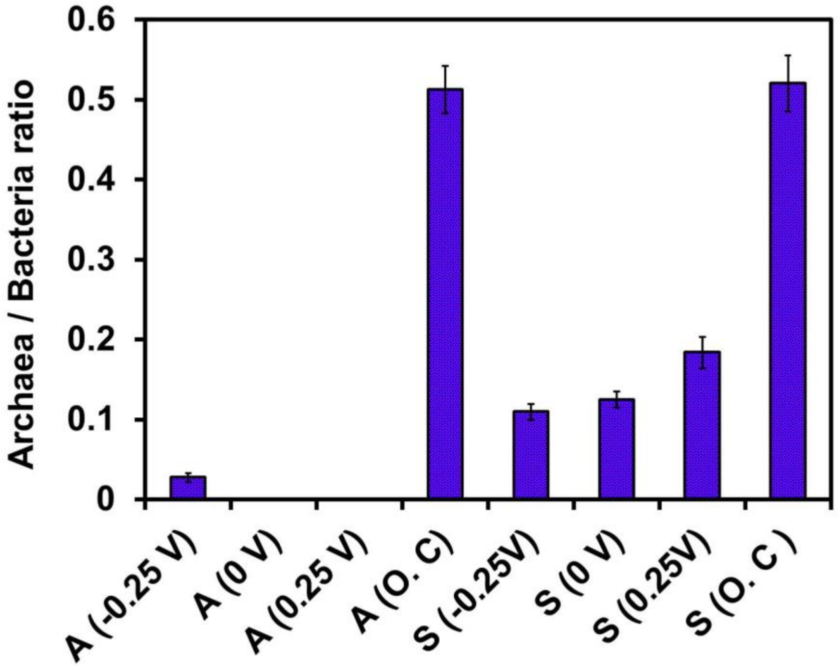

Figure S5 The ratio of Archaea to Bacteria in the anode and suspension samples of the different SAP (-0.25, 0 and 0.25 V) and open circuit (O.C) reactors. “A” and “S” correspond to the anode and suspension.

Standard Gibbs Free Energy Changes for Electricity and Methane Generating Reactions

Electricity generating reactions

$\Delta G^{\circ\prime}$ was calculated using the equation $\Delta G^{\circ\prime} = - nF(E_{anode} - E^{\circ\prime}_{substrate})$ [11]. Where $\Delta G^{\circ\prime}$ (kJ/mol) is the Gibbs free energy at standard biological conditions (i.e. reactant and products at 1 M or 1 atm, 298 K, and pH 7), n is the number of electrons transferred, F is the Faradays constant (96,485 C/mol e^-), and E_{anode} (V) and $E^{\circ\prime}_{substrate}$ (V) are the anode potential and the standard biological redox potential of the substrate. $E^{\circ\prime}_{substrate}$ was calculated using equation $E^{\circ\prime}_{substrate} = -\Delta G^{\circ}_{r} / nF$ [14] and values from Ref. [15].

Reactions	$\Delta G^{\circ\prime}$ (kJ/mol)		
	**(-0.25 V)**	**0 V**	**(0.25 V)**
$CH_3COO^- + 4H_2O \longrightarrow 2HCO_3^- + 9H^+ + 8e^-$	-21.6	-214.6	-407.6
$H_2 \longrightarrow 2H^+ + 2e^-$	-31.5	-79.7	-128
$HCOOH + H_2O \longrightarrow HCO_3^- + 2H^+ + 2e^-$	-46.1	-94.4	-142.6

CH_4 generating reactions

Standard Gibbs free energy changes of CH_4 generating reactions were obtained from [16]

	$\Delta G^{\circ\prime}$ (kJ/mol)
$CH_3COO^- + 4H_2O \longrightarrow HCO_3^- + CH_4$	-31 kJ/mol
$4H_2 + HCO_3^- + H^+ \longrightarrow CH_4 + 3H_2O$	-136 kJ/mol
$4\ HCOOH + H^+ \longrightarrow 3HCO_3^- + 2H_2O + CH_4$	-130 kJ/mol

Theoretical Distribution of Electrons from Propionate Oxidation to Various Electron Sinks

Conversion of propionate concentration from mM to milli electron (me^-) equivalents

Conversion of propionate concentration from mM to me^- equivalents = Propionate concentration (mM)×volume of the reactor (Litre)×moles of electrons/ mole of propionate.

For example 35.8 mM of propionate used in this study is equivalent to 20 me^- equivalents, calculated as follows:

$$35.8 \times 0.04 \times 14 = 20\ me^- \text{ equivalents}$$

Electrons utilized for biomass synthesis

The fraction of electrons from the donor substrate that is utilized for biomass synthesis is represented by f^0_s [17]. $f^0_s = 0.05$ for *G. sulfurreducens*, as representative of exoelectrogens [18,19]; $f^0_s = 0.1$ for fermenters and $f^0_s = 0.08$ for hydrogenotrophic methanogens [17]. The electrons utilized for the different microbial groups was calculated by multiplying the substrate concentration in me^- equivalent with the f^0_s values of the corresponding organisms.

For example, the electrons utilized for propionate fermenters biomass (2) was calculated by multiplying 20 me^- equivalent by the f^0_s value (0.1) for propionate fermenters (Supplementary Figure S6).

Predicted electron distribution to various electron sinks (Supplementary Figure S6)

Two possible pathways of propionate oxidation could occur in the anode of MECs. Pathways 1 and 2 involve a microbial partnership between propionate fermenting bacteria and fermentation

products (acetate, formate, and H_2) consumers (i.e. exoelectrogens and hydrogenotrophic methanogens). In pathways 1 and 2, propionate is oxidized to acetate and hydrogen (pathway 1) or formate (pathway 2). The acetate produced could then be oxidized by exoelectrogens to produce current. The hydrogen and formate will be utilized by hydrogenotrophic methanogens to produce methane or by exolectrogens to produce current.

Pathway 1 (all values in bracket are presented as me⁻ equivalents):
Propionate (20) undergoes oxidation by propionate fermenters yielding biomass (2), H_2 (7.72) and acetate (10.28). H_2 (7.72) generated from propionate fermentation is utilized by hydrogenotrophic methanogens yielding biomass (0.6) and methane (7.12), or it could be oxidized by *G. sulfurreducens* yielding biomass (0.4) and current (7.32). Also, the acetate (10.28) generated from propionate fermentation is oxidized by *G. sulfurreducens* yielding biomass (0.6) and current (9.68). If hydrogenotrophic methanogens outcompete exolectrogens for hydrogen then 36% of the electrons in propionate will be lost to methane; and the maximum flow of electrons from propionate to current will be 9.68 me^{-} equivalents (i.e. 48% of the electrons in propionate) from acetate oxidation alone.

Pathway 2 (all values in bracket are presented as me⁻ equivalents):
Propionate (20) undergoes oxidation by propionate fermenters yielding biomass (2), formate (7.72), and acetate (10.28). Formate (7.72) generated from propionate fermentation is utilized by hydrogenotrophic methanogens yielding biomass (0.6) and methane (7.12), or it could be oxidized by *G. sulfurreducens* yielding biomass (0.4) and current (7.32). Also, Acetate (10.28) generated from propionate fermentation is oxidized by *G. sulfurreducens* yielding biomass (0.6)

and current (9.68). If hydrogenotrophic methanogens outcompete exolectrogens for formate then 36% of the electrons in propionate will be lost to methane; and the maximum flow of electrons from propionate to current will be 9.68 me^- equivalents (i.e. 48% of the electrons in propionate) from acetate oxidation alone.

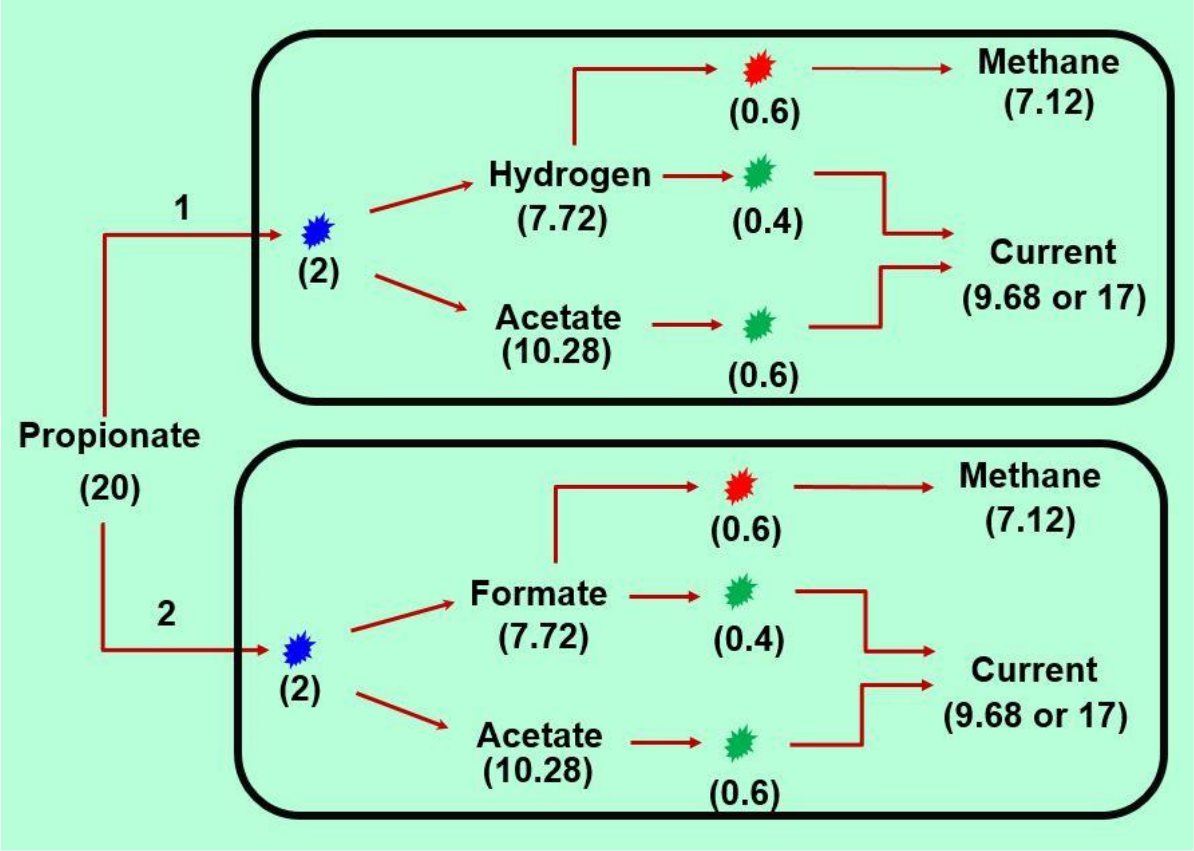

Figure S6 Possible theoretical pathways of electron flow in the anode of MECs fed with propionate (20 me^- equivalents =36 mM). All values in bracket are presented as me^- equivalents. Blue represents fermenters, red represents methanogens and green represents exoelectrogens. Figure S4 was created by Ananda Rao Hari.

References

1 Ambler, J. R. & Logan, B. E. Evaluation of stainless steel cathodes and a bicarbonate buffer for hydrogen production in microbial electrolysis cells using a new method for measuring gas production. *Int. J. Hydrogen. Energy.* **36**, 160-166 (2011).

2 Lee, H. S., Torres, C. I., Parameswaran, P. & Rittmann, B. E. Fate of H2 in an upflow single-chamber microbial electrolysis cell using a metal-catalyst-free cathode. *Environ. Sci. Technol.* **43**, 7971-7976 (2009).

3 Takahashi, S., Tomita, J., Nishioka, K., Hisada, T. & Nishijima, M. Development of a prokaryotic universal primer for simultaneous analysis of Bacteria and Archaea using next-generation sequencing. *PloS one.* **9**, e105592 (2014).

4 Caporaso, J. G. *et al.* QIIME allows analysis of high-throughput community sequencing data. *Nature Met.* **7**, 335-336 (2010).

5 Edgar, R. C. Search and clustering orders of magnitude faster than BLAST. *Bioinformatics.* **26**, 2460-2461 (2010).

6 Caporaso, J. G. *et al.* PyNAST: a flexible tool for aligning sequences to a template alignment. *Bioinformatics.* **26**, 266-267 (2010).

7 Wang, Q., Garrity, G. M., Tiedje, J. M. & Cole, J. R. Naive Bayesian classifier for rapid assignment of rRNA sequences into the new bacterial taxonomy. *Appl. Environ. Microbiol.* **73**, 5261-5267 (2007).

8 Lozupone, C. & Knight, R. UniFrac: a new phylogenetic method for comparing microbial communities. *Appl. Environ. Microbiol.* **71**, 8228-8235 (2005).

9 Torres, C. I. *et al*. Selecting anode-respiring bacteria based on anode potential: phylogenetic, electrochemical, and microscopic characterization. *Environ. Sci. Technol.* **43**, 9519-9524 (2009).

10 Commault, A. S., Lear, G., Packer, M. A. & Weld, R. J. Influence of anode potentials on selection of *Geobacter* strains in microbial electrolysis cells. *Bioresour. Technol.* **139**, 226-234 (2013).

11 Zhu, X. *et al.* Microbial community composition is unaffected by anode potential. *Environ. Sci. Technol.* **48**, 1352-1358 (2014).

12 Villano, M., Ralo, C., Zeppilli, M., Aulenta, F. & Majone, M. Influence of the set anode potential on the performance and internal energy losses of a methane-producing microbial electrolysis cell. *Bioelectrochem.* **107**, 1-6 (2016).

13 Parameswaran, P., Torres, C. I., Lee, H. S., Krajmalnik-Brown, R. & Rittmann, B. E. Syntrophic interactions among anode respiring bacteria (ARB) and Non-ARB in a biofilm anode: electron balances. *Biotechnol. Bioeng.* **103**, 513-523 (2009).

14 Logan, B. E. *et al.* Microbial fuel cells: methodology and technology. *Environ. Sci. Technol.* **40**, 5181-5192 (2006).

15 Heijnen, J. J. Bioenergetics of Microbial Growth Encyclopedia of Bioprocess Technology. John Wiley & Sons, Inc. 162 (2002).

16 Thauer, R. K., Jungermann, K. & Decker, K. Energy conservation in chemotrophic anaerobic bacteria. *Bacteriol. Rev.* **41**, 100 (1977).

17 Rittmann, B. E. & McCarty, P. L. *Environmental biotechnology*. (McGraw-Hill New York, 2001).

18 Bond, D. R. & Lovley, D. R. Electricity production by Geobacter sulfurreducens attached to electrodes. *Appl. Environ. Microbiol.* **69**, 1548-1555 (2003).

19 Esteve-Núñez, A., Rothermich, M., Sharma, M. & Lovley, D. Growth of Geobacter sulfurreducens under nutrient-limiting conditions in continuous culture. *Environ. Microbiol.* **7**, 641-648 (2005).
